# Reirradiation for diffuse intrinsic pontine glioma: prognostic radiomic factors at progression

**DOI:** 10.1007/s00066-024-02241-7

**Published:** 2024-05-15

**Authors:** Dominik Wawrzuta, Marzanna Chojnacka, Monika Drogosiewicz, Katarzyna Pędziwiatr, Bożenna Dembowska-Bagińska

**Affiliations:** 1 Department of Radiation Oncology, Maria Sklodowska-Curie National Research Institute of Oncology, Wawelska 15B, 02-034 Warsaw, Poland; 2 Department of Oncology, Children’s Memorial Health Institute, Al. Dzieci Polskich 20, 04-730 Warsaw, Poland

**Keywords:** Diffuse intrinsic pontine glioma, Diffuse midline glioma, Reirradiation, Radiomics, Radiotherapy, Magnetic resonance imaging

## Abstract

**Purpose:**

Diffuse intrinsic pontine glioma (DIPG) is a lethal pediatric brain tumor. Radiation therapy (RT) is the standard treatment, with reirradiation considered in case of progression. However, the prognostic factors for reirradiation are not well understood. This study aims to investigate the outcomes of DIPG patients undergoing reirradiation and identify clinical and radiomic prognostic factors.

**Methods:**

We conducted a retrospective analysis of patients with DIPG who underwent reirradiation at our institution between January 2016 and December 2023. Using PyRadiomics, we extracted radiomic features of tumors at the time of progression from FLAIR MRI images and collected clinical data. We used the least absolute shrinkage and selection operator (lasso) for Cox’s proportional hazard model with leave-one-out cross-validation to select optimal prognostic factors for survival after reirradiation.

**Results:**

The study included 18 patients who underwent reirradiation at first progression, receiving a total dose of 20 Gy or 24 Gy in 2‑Gy fractions. Reirradiation was well tolerated, with no severe toxicity. Most patients (78%) showed neurological improvement after treatment. Median survival after progression was 29.2 weeks. The Cox model demonstrated a concordance of 0.81 (95% CI: 0.75–0.88), revealing that tumor sphericity and structural gray-level heterogeneity in FLAIR MRI images were associated with longer survival of reirradiated patients.

**Conclusion:**

Reirradiation is a safe and effective approach for patients with DIPG. MRI-based radiomic models could be helpful in predicting survival after reirradiation.

## Introduction

### Background

Diffuse intrinsic pontine glioma (DIPG) is a lethal pediatric brain tumor that mainly affects school-aged children [1]. Despite abundant research involving chemotherapeutics and targeted therapies, radiation therapy remains the cornerstone of treatment [2]. Conventionally fractionated focal radiotherapy up to a total dose of 54 Gy is the standard of care for DIPG. However, noninferior hypofractionated approaches of 39 Gy in 13 or 44.8 Gy in 16 fractions are particularly recommended in case of rapid disease progression [3, 4]. The primary objective of radiation therapy is to alleviate neurological symptoms and improve quality of life and overall survival (OS) [5].

Reirradiation becomes a viable therapeutic option at the time of progression. Standard treatment involves administering total doses from 20 to 30 Gy in 1.8–2.Gy fractions [6, 7]. About 80% of patients experience clinical improvement following reirradiation, which alleviates symptoms and improves OS [7, 8]. Higher fraction and total doses have not demonstrated superior efficacy; rather, they have increased the risk of toxicity, as evidenced by pons necrosis reported after exposure to 30 Gy in 10 fractions [8, 9]. Due to the rarity of DIPG, there are still few data regarding the effects of reirradiation. The most extensive meta-analysis published to date included seven studies, encompassing only 90 reirradiated patients [8].

Although reirradiation is a crucial treatment strategy, prognostic factors remain poorly explored. Currently, the interval between initial radiation therapy and the onset of disease progression is the only established prognostic factor [7]. The emergence of magnetic resonance imaging (MRI)-based radiomics in the context of brain tumors can potentially help to discover novel variables that influence treatment outcomes [10]. Although the usefulness of MRI-based radiomics in modeling DIPG outcomes has been established for newly diagnosed children [11], their applicability to reirradiated patients needs to be explored.

### Aim of the study

This study aims to identify clinical and MRI-based prognostic radiomic factors for reirradiated children with DIPG.

## Methods

### Study cohort

We enrolled a cohort of 18 patients under the age of 19 years with first DIPG progression after upfront treatment. All children had histopathologically confirmed DIPG in their initial diagnosis. The decision to use a second radiotherapy was reached through consensus between pediatric oncologists and radiation oncologists. All patients underwent reirradiation in the Department of Radiotherapy at Maria Sklodowska-Curie National Research Institute of Oncology in Warsaw between January 2016 and December 2023. They initially received a total radiation dose of 54 Gy delivered in 1.8-Gy fractions, and demonstrated a minimum 3‑month period of progression-free survival (PFS). For reirradiation, a total dose of 20 Gy or 24 Gy was administered in 2‑Gy fractions. Twelve patients received first-line systemic treatment.

### Clinical analysis

Patients’ neurological symptoms were monitored daily to evaluate the effectiveness and toxicity of the treatment. Toxicity was assessed using the Common Terminology Criteria for Adverse Events (CTCAE) scale. Follow-up care was provided in the specialized pediatric oncology department following completion of radiotherapy. We established a control group of 25 patients who also met the eligibility criteria for reirradiation to evaluate and compare survival outcomes in both groups. Following initial standard-dose radiation therapy, these patients experienced at least 3 months without disease progression. However, at progression, their families chose supportive care without second irradiation. We compared the clinical characteristics of reirradiated and non-reirradiated patients using the chi-square test and the Wilcoxon test for categorical and continuous variables, respectively. Additionally, we employed the Westenberg–Mood median test to assess medians. Finally, we estimated survival rates using the Kaplan–Meier method and evaluated significance between the two groups using the log-rank test, which was facilitated by the survminer R package (version 0.4.9; R foundation, Vienna, Austria) [12].

### Radiomic features

We performed the radiomic analysis in the cohort of patients who underwent reirradiation. MRI scans of the brain at the time of progression were taken with a 1.5 T magnetic resonance scanner (Siemens Healthineers, Erlangen, Germany). The slice thickness was 5 mm in the T2-weighted fluid-attenuated inversion recovery (T2 FLAIR) sequences and the matrix size range 300–500 × 300–500. We manually delineated tumor volumes using FLAIR MRI images with the help of 3D Slicer software (version 5.0.3) [13] to extract the radiomic features of tumors in the reirradiated group. All contouring was performed by DW and validated by MC. In case of disagreement, the final tumor boundaries were established by consensus.

Subsequently, we extracted tumor volumes and calculated their radiomic characteristics from normalized FLAIR MRI sequences using the PyRadiomics Python package (version 3.1.0) [14]. We generated a comprehensive set of radiomic features, encompassing first-order features, shape features, gray-level cooccurrence matrix (GLCM) features, gray-level size zone matrix (GLSZM) features, gray-level run length matrix (GLRLM) features, neighboring gray tone difference matrix (NGTDM) features, and gray-level dependence matrix (GLDM) features, for a total of 102 variables.

### Prognostic model

We employed the least absolute shrinkage and selection operator (lasso) for Cox’s proportional hazard model, implemented in the glmnet R package (version 4.1.4) [15], to identify the most prognostic features for survival. In our prognostic model before regularization, we incorporated radiomic, clinical, and demographic variables, including sex, age at the first radiotherapy, age at the second radiotherapy, the use of systemic therapy, survival after the first radiotherapy, survival after the second radiotherapy, and the time between the first and second treatments. All predictors were standardized. We used the leave-one-out cross-validation method to find the lambda value (the parameter governing the amount of regularization) and mitigate bias and the risk of overfitting associated with a small sample size [16]. Optimal features were selected based on the lambda value with the lowest error across all repetitions. After that, we assessed the collinearity of the chosen variables. In addition, we created univariate models for each variable to cross-check their individual impact on survival with the impact estimated in multivariate regression.

## Results

### Patient characteristics

We analyzed 18 patients who underwent reirradiation between January 2016 and December 2023. The total radiation dose was 20 Gy in 16 patients and 24 Gy in 2 patients. The 24 Gy dose was administered to patients who showed progression-free survival (PFS) over 1 year from primary radiation therapy. Detailed patient characteristics are presented in Table 1. During treatment, 14 out of the 18 patients (78%) showed neurological improvement. No adverse event episodes of grade > 2 toxicity were observed. Four patients presented mild symptoms indicative of increased intracranial pressure. The median survival time from progression was 29.2 weeks. No significant differences were observed among the subgroups receiving systemic therapy. The median survival for patients receiving temozolomide was 30.0 weeks, for those receiving sirolimus it was 27.6 weeks, and for patients undergoing only radiotherapy it was 29 weeks.

**Table 1:** Characteristics of patients

Characteristics of patients			
	Reirradiated (*N* = 18)	Non-reirradiated (*N* = 25)	*p*-value
*Gender*			
Male	9 patients (50%)	14 (56%)	0.94
Female	9 patients (50%)	11 (44%)	
*Age*			
Median age at the diagnosis	7.5 years	7.1 years	0.72
Median age at the progression	9.1 years	7.8 years	0.96
*First-line RT regimen*			
54 Gy in 1.8-Gy fractions	18 patients (100%)	22 patients (88%)	0.36
44.8 Gy in 2.8-Gy fractions	0 patients (0%)	3 patients (12%)	
*Neurological symptoms at diagnosis*			
Ataxia	12 patients (67%)	16 patients (64%)	0.55
Long tract signs	5 patients (28%)	11 patients (44%)	
Cerebral neuropathy	9 patients (50%)	21 patients (84%)	
*First-line RT effectiveness*			
Neurological improvement	18 patients (100%)	25 patients (100%)	1
Median progression-free survival	43.7 weeks	38.3 weeks	0.18
*First-line RT toxicity*			
Mild symptoms of increased intracranial pressure (grade 1)	2 patients (11%)	3 patients (12%)	1
*Systemic therapy regimen before progression*			
Temozolomide	8 patients (44%)	5 patients (20%)	0.51
Sirolimus	5 patients (28%)	8 patients (32%)	
Nivolumab	1 patient (6%)	1 patient (4%)	
Only RT	6 patients (33%)	11 patients (44%)	
*Second-line RT regimen*			
20 Gy in 2‑Gy fractions	16 patients (89%)	–	–
24 Gy in 2‑Gy fractions	2 patients (11%)	–	
*Neurological symptoms at progression*			
Ataxia	12 patients (67%)	14 patients (64%)	0.33
Long tract signs	9 patients (50%)	19 patients (76%)	
Cerebral neuropathy	8 patients (44%)	21 patients (84%)	
*Second-line RT effectiveness*			
Neurological improvement	14 patients (78%)	–	–
*Second-line RT toxicity*			
Mild symptoms of increased intracranial pressure (grade 1)	4 patients (22%)	–	–
*Survival*			
Median survival after diagnosis	79.1 weeks	46.1 weeks	< 0.01
Median survival after progression	29.2 weeks	7.1 weeks	< 0.01
*Genetic alterations*			
Proven *H3K27* mutation	9 patients (50%)	11 patients (44%)	0.94

**Fig. 1:**
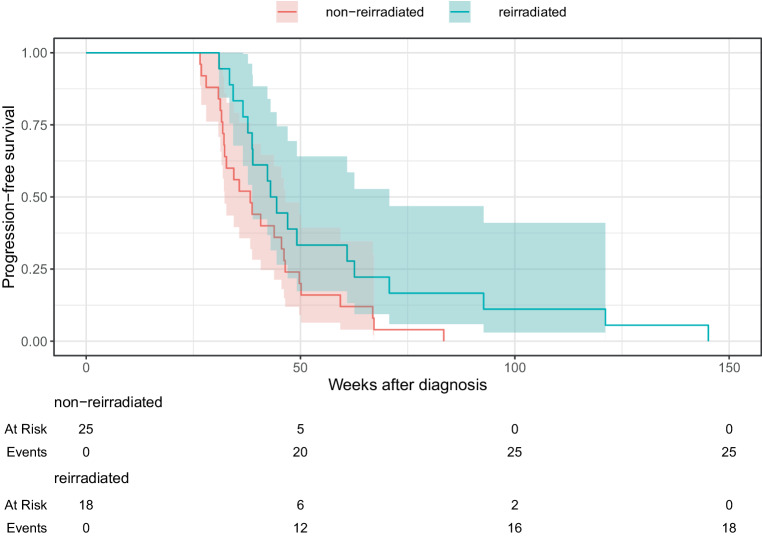
Progression-free survival after diagnosis

**Fig. 2:**
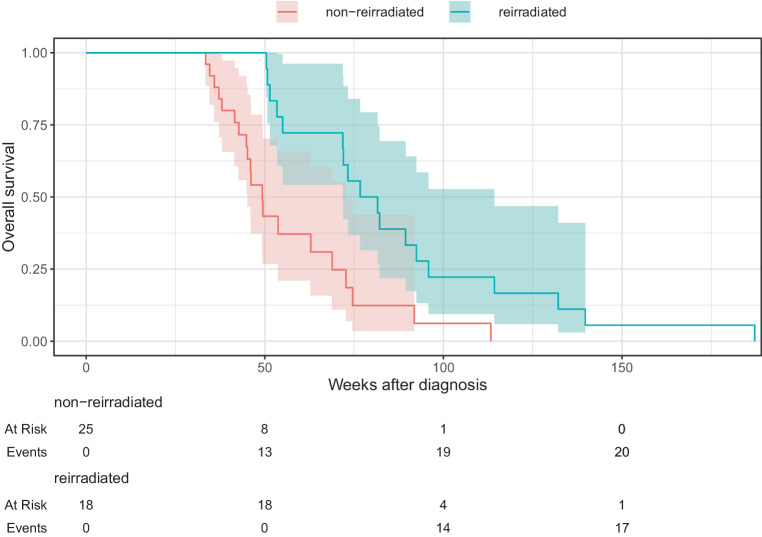
Overall survival after diagnosis

**Fig. 3:**
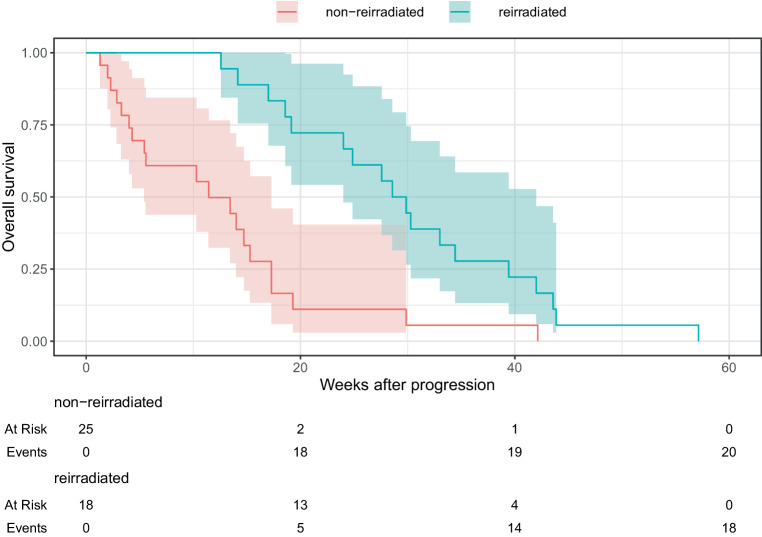
Overall survival after progression

We compared the reirradiated group with a cohort of 25 non-reirradiated patients with DIPG who were potentially eligible for reirradiation to evaluate the efficacy of reirradiation, as their survival time exceeded 3 months after initial radiation therapy. There were no statistically significant differences between the groups regarding gender, age, first-line RT regimen, neurological symptoms at diagnosis and progression, first-line RT effectiveness and toxicity, PFS, and systemic therapy use, as illustrated in Table 1 and Fig. 1. The only statistically significant difference was observed in survival. The reirradiated group exhibited a significantly longer median survival time from diagnosis (79.1 weeks) and progression (29.2 weeks) compared to the non-reirradiated group (46.1 weeks and 7.1 weeks, respectively). The survival disparity between the groups was statistically significant according to the log-rank test (*p* < 0.01). The Kaplan–Meier survival curves are shown in Fig. 2 and 3.

### Prognostic factors

We identified radiomic variables as key predictors of survival. Table 2 summarizes the coefficients of the multivariate Cox regression model with a robust concordance index of 0.81 (95% confidence interval [CI]: 0.75–0.88). Lasso gave non-zero coefficients to shape_Flatness and glszm_SizeZoneNonUniformityNormalized. Prognostic factors derived from radiomic analysis can be categorized into two groups. The first group is tumor sphericity represented by the feature shape_Flatness (HR = 0.48 [95% CI: 0.26–0.87], *p* = 0.02). High tumor sphericity means a low discrepancy between the longest and shortest diameters (high shape_Flatness value). The second group refers to the distribution of gray levels within FLAIR MRI images, encompassing the variable glszm_SizeZoneNonUniformityNormalized (HR = 0.30 [95% CI: 0.13–0.69], *p* < 0.01). A low value of gray-level nonuniformity indicates more homogeneity in intensity values.

**Table 2:** Summary of the multivariate Cox model

Explanatory variable	Hazard ratio (95% CI)	*p*-value
Shape_Flatness	0.48 (0.26–0.87)	0.02
Glszm_SizeZoneNonUniformityNormalized	0.30 (0.13–0.69)	< 0.01

Table 3 presents the radiomic features selected by lasso for Cox’s proportional hazard model, along with their hazard ratios (HR) derived from the univariate Cox models. The coefficients from the univariate regressions remain consistent in sign and magnitude with those obtained in the multivariate regression, suggesting low correlations between predictors. Specifically, shape_Flatness (HR = 0.61 [95% CI: 0.35–1.06], *p* = 0.08) and glszm_SizeZoneNonUniformityNormalized (HR = 0.43 [95% CI: 0.22–0.85], *p* = 0.02) exhibited negative associations with the hazard ratio.

**Table 3:** Summary of univariate Cox models

Explanatory variable	Hazard ratio (95% CI)	*p*-value
Shape_Flatness	0.61 (0.35–1.06)	0.08
Glszm_SizeZoneNonUniformityNormalized	0.43 (0.22–0.85)	0.02

**Fig. 4:**
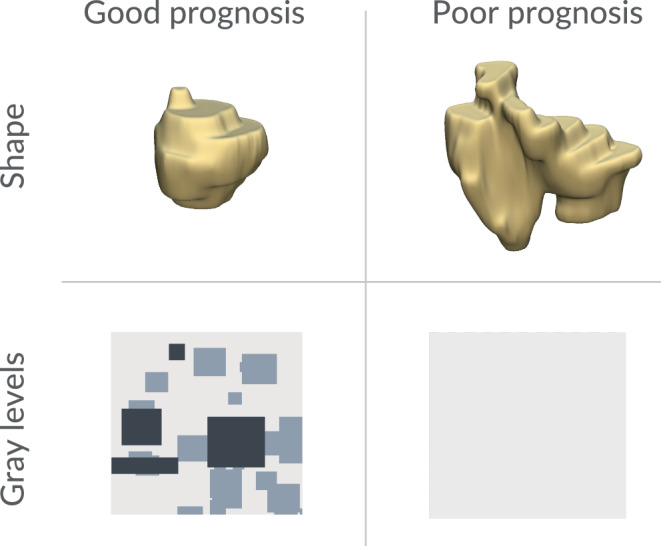
Characteristics of tumors with good and poor prognoses

Finally, we interpreted the results of the multivariate Cox analysis and prepared illustrative examples of tumors with divergent prognoses based on our model, as shown in Fig. 4. Higher tumor sphericity is associated with a better prognosis. Regarding gray-level distribution, tumors featuring nonuniform gray-level patterns demonstrate a more favorable prognosis than tumors with a homogeneous structure.

## Discussion

### Patient characteristics

Consistent with previous reports, our cohort’s median age at the time of disease diagnosis was almost 8 years and the genders were equally distributed [1]. Following initial radiation therapy, the patients received continuous care in the department of pediatric oncology. Follow-up care was provided in the same center, ensuring that patients with disease progression underwent consistent evaluations of eligibility for reirradiation based on uniform criteria. The median PFS after initial treatment in the reirradiated group was 9 months, similar to the time reported by Janssens et al. [7]. However, other studies reported that time from the start of the first radiation therapy to reirradiation exceeded 12 months [6, 17]. This discrepancy can be attributed to dissimilar inclusion criteria adopted in different centers. Compared to our cohort, some radiotherapy departments demand a more prolonged survival period for reirradiation [8].

### Reirradiation in DIPG

Patients who responded well to their initial irradiation and had a minimum survival period of 3 months were eligible for reirradiation. At the time of progression, reirradiation was considered after a thorough discussion with the patients’ parents, who were informed about the potential benefits and associated risks. However, we did not collect surveys on the reasons for choosing or refusing radiotherapy by parents. During reirradiation, we adhered to the standard of care, which recommends delivering a total dose of 20 Gy distributed in 10 fractions to the clinical target volume (CTV), which includes the gross tumor volume (GTV) with a 5-mm margin, following the recommendations of the SIOPE working group [7]. However, for patients who had survived for at least 12 months after their initial treatment, we proposed an elevated dose of 24 Gy distributed over 12 fractions because, after this period, the brainstem recovers [18, 19].

Our study reported a 78% rate of neurological improvement, aligning with the 77% rate reported by Janssens et al. [6]. We did not observe any grade > 2 toxicity, which is consistent with previous research suggesting that severe toxicity may be associated with fractional doses higher than 3 Gy and a total dose of 30 Gy [8, 9]. Although a direct comparison of efficacy between a total dose of 20 Gy and 24 Gy in our cohort was not feasible due to the limited number of patients receiving the higher dose, it is essential to highlight the absence of increased toxicity. The existing literature lacks precise guidance on who should receive the higher dose, with current publications offering only limited differentiation between these dose regimens. However, Chavaz et al. [20] reported that doses greater than 20 Gy may result in better outcomes regarding ataxia.

### Prognostic factors in reirradiated DIPG patients

In our multivariate analysis, clinical variables did not emerge as significant predictors, and only radiomic features were found to explain survival after reirradiation. This finding contrasts with previous research by Janssens et al. [7], who identified the time between the first and second irradiations as a prognostic factor for reirradiated patients with DIPG. Children in our cohort who had a longer time to progression had also experienced extended survival after reirradiation. However, statistical significance was not attained, most likely due to our limited sample size.

Most studies on prognostic radiological factors in DIPG have focused on patients at initial diagnosis. However, the evolving tumor structure after the first irradiation and during progression requires a distinct analysis for reirradiated patients. Our findings are consistent with those of Tam et al. [11], who focused on patients undergoing initial irradiation. They observed that heterogeneous tumor pixel intensity or texture correlated with a better prognosis. Although the exact explanation remains elusive, in reirradiated patients, this finding can be attributed to the presence of heterogeneities resulting from necrosis after initial irradiation. These necrotic changes suggest increased tumor radiosensitivity, which may lead to a more favorable response to second radiation therapy. However, additional research, including histopathological postmortem studies, could yield valuable data to elucidate this association.

Our study revealed that spherical tumors exhibit a more favorable prognosis than tumors whose longest and shortest diameters differ significantly. We observed that non-spherical tumors in our cohort were predominantly cases involving extrapontine tumor extensions, often in regions of the thalamus or cerebellar peduncles. This situation is usually associated with advanced disease. This finding aligns with earlier literature on initially irradiated DIPG patients, which associated extrapontine extensions with less favorable survival [21, 22].

### Limitations and future directions

Although our study represents one of the largest published cohorts of patients with reirradiated DIPG, the sample size of 18 remains relatively small, which presents challenges in drawing robust statistical conclusions. We tried to minimize instability and the risk of overfitting using leave-one-out cross-validation, a suitable method for small datasets [23]. However, we recommend conducting a multi-institutional analysis of prognostic factors that would incorporate genetic factors. Genetic analysis was omitted from our study due to the inaccessibility of specific genetic data for some patients within our cohort. Exploring the connection between radiomic and genetic characteristics could significantly enhance our understanding of DIPG prognosis. The potential impact of tumor heterogeneity on survival deserves attention in future clinical trials. These trials could explore personalized radiation therapy strategies, particularly by examining the use of nonuniform dose distributions in necrotic or hypoxic regions. Furthermore, analyzing radiomic data from non-reirradiated patients with DIPG may reveal subgroups with favorable survival outcomes without reirradiation.

## Conclusion

Reirradiation is a safe and effective treatment method for patients with progressive DIPG. Multiparametric MRI-based radiomic models could help predict survival in DIPG after reirradiation. We found that spherical tumors with nonhomogeneous gray-value distributions have the best prognosis after reirradiation.
